# Complexities of deep learning-based undersampled MR image reconstruction

**DOI:** 10.1186/s41747-023-00372-7

**Published:** 2023-10-04

**Authors:** Constant Richard Noordman, Derya Yakar, Joeran Bosma, Frank Frederikus Jacobus Simonis, Henkjan Huisman

**Affiliations:** 1 Diagnostic Image Analysis Group, Department of Medical Imaging, Radboud University Medical Center, Nijmegen, 6525 GA The Netherlands; 2 Medical Imaging Center, Departments of Radiology, Nuclear Medicine and Molecular Imaging, University Medical Center Groningen, Groningen, 9700 RB The Netherlands; 3 Magnetic Detection and Imaging Group, Technical Medical Centre, University of Twente, Enschede, 7522 NB The Netherlands; 4 Department of Circulation and Medical Imaging, Norwegian University of Science and Technology, Trondheim, 7030 Norway

**Keywords:** Algorithm, Artificial intelligence, Deep learning, Image processing (computer-assisted), Magnetic resonance imaging

## Abstract

Artificial intelligence has opened a new path of innovation in magnetic resonance (MR) image reconstruction of undersampled k-space acquisitions. This review offers readers an analysis of the current deep learning-based MR image reconstruction methods. The literature in this field shows exponential growth, both in volume and complexity, as the capabilities of machine learning in solving inverse problems such as image reconstruction are explored. We review the latest developments, aiming to assist researchers and radiologists who are developing new methods or seeking to provide valuable feedback. We shed light on key concepts by exploring the technical intricacies of MR image reconstruction, highlighting the importance of raw datasets and the difficulty of evaluating diagnostic value using standard metrics.

**Relevance statement** Increasingly complex algorithms output reconstructed images that are difficult to assess for robustness and diagnostic quality, necessitating high-quality datasets and collaboration with radiologists.

**Key points**

• Deep learning-based image reconstruction algorithms are increasing both in complexity and performance.

• The evaluation of reconstructed images may mistake perceived image quality for diagnostic value.

• Collaboration with radiologists is crucial for advancing deep learning technology.

**Graphical Abstract:**

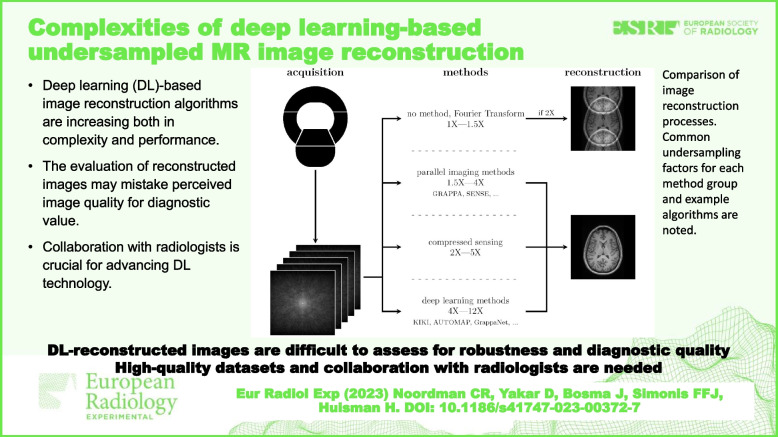

## Background

Magnetic resonance (MR) is a popular modality in medical imaging for its versatility, nonionizing radiation, and good soft-tissue contrast. However, its relatively long acquisition times, incurring high costs and patient discomfort, has led to a flurry of research to improve imaging speed without compromising image quality.

MR data is acquired in the spatial frequency domain, referred to as k-space. Applying an inverse Fourier transform gives a reconstructed spatial image. Sampling less of the k-space decreases scan time but may introduce aliasing. The Nyquist criterion specifies a minimum sampling density required to avoid detrimental wrap-around artifacts during reconstruction, and sampling under this minimum is considered undersampling. The acceleration factor denotes the extent of undersampling; an acceleration factor of 4 corresponds to sampling 25% of the lines in k-space [1–3].

Undersampled acquisitions are traditionally reconstructed using parallel imaging methods with multiple receive coils, such as sensitivity encoding (SENSE) or the generalized autocalibrating partial parallel acquisition (GRAPPA) techniques [1, 2]. However, parallel imaging reconstructions suffer a signal-to-noise ratio (SNR) loss at least proportional to the square root of the reduction in scan time [2]. Compressed sensing is an alternative, an iterative optimization process that reconstructs using a priori information known as sparsity [3]. Compressed sensing-based reconstructions suffer from blurring and ringing artifacts, which are considered not as detrimental to diagnostic quality [4].

Advances in artificial intelligence techniques and development in computational infrastructures have led to machine learning techniques becoming viable candidates to aid in medical image reconstruction. Deep learning (DL)-based image reconstruction methods use reference data as a priori information for learning features to exploit the similarities in patients’ anatomy, and proposed methods have shown superior performance compared to non-DL-based solutions [5–9]. Moreover, some DL methods integrate traditional iterative algorithms, such as compressed sensing, to further improve their performance [10–12]. Public datasets, such as the fastMRI dataset, support the effort by providing a consistent benchmark for new machine-learning approaches in MR image reconstruction [13].

Radiologists need to remain up to speed with the latest advances in image reconstruction. A basic understanding of the research and development is crucial to providing researchers with valuable feedback for further improvement. Furthermore, radiologists need to become acquainted with novel artifacts exclusive to machine-assisted reconstructions, should these new methods make it into clinical use. This narrative review aims to introduce DL-based image reconstruction and provide insight into researchers’ current challenges. Following an introductory section on DL-based MRI reconstruction networks, we explore some technical topics specific to MR image reconstruction networks, which received substantial attention in the literature, followed by topics on the training and evaluation of these networks.

## DL-based MR image reconstruction methods

**Fig. 1:**
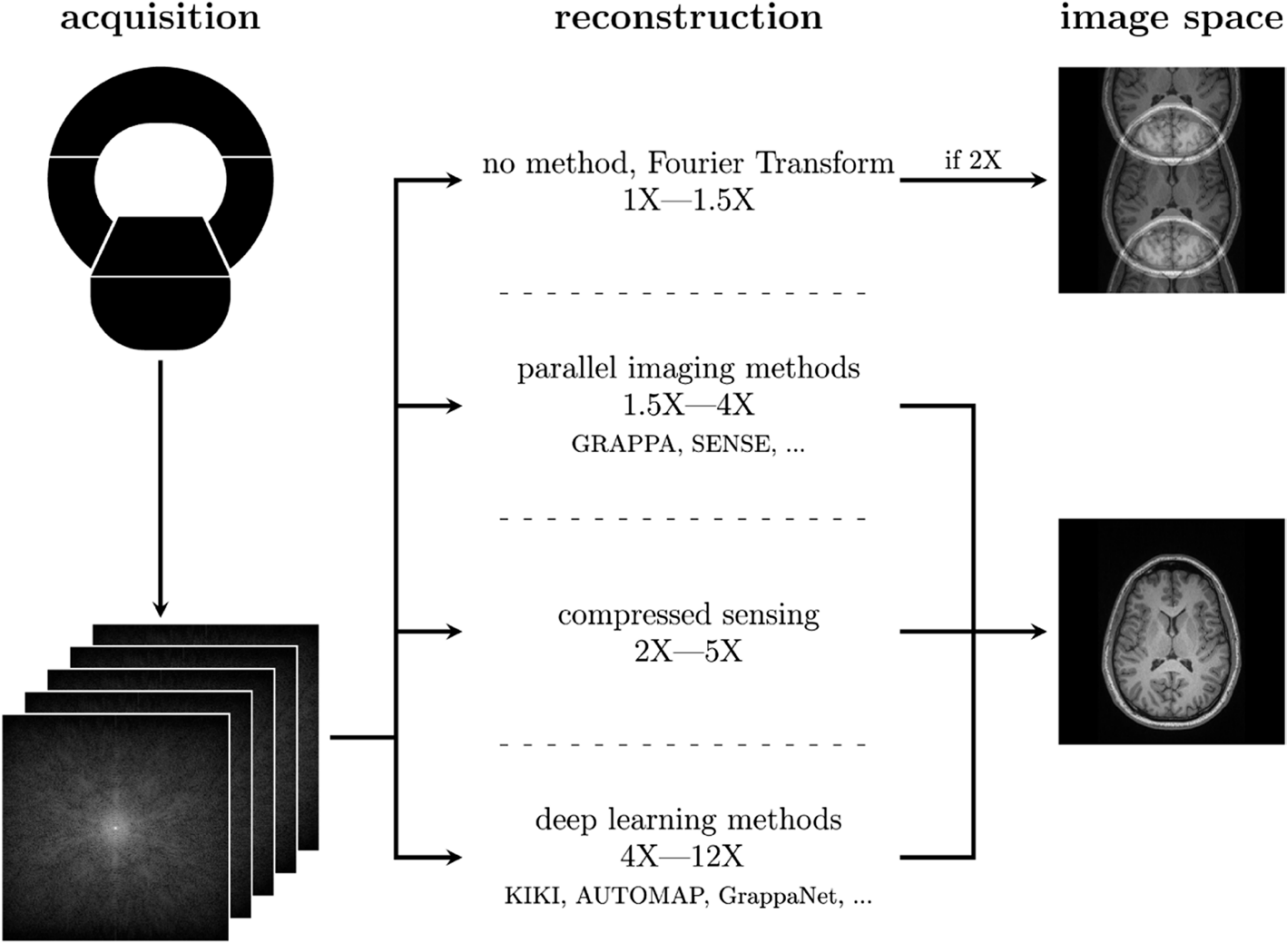
Comparison of image reconstruction processes. Common undersampling factors for each method group and a few example algorithms are noted. If no method is used, a simple Fourier transform results in an aliased image if the acquisition is undersampled. Parallel imaging reconstruction methods, such as the generalized autocalibrating partial parallel acquisition (GRAPPA) or sensitivity encoding (SENSE) algorithms, can produce acceptable results up to undersampling rates of 4. Compressed sensing can achieve similar results with increased levels of undersampling. Deep learning methods, such as KIKI, automated transform by manifold approximation (AUTOMAP), and GrappaNet, have shown the potential to achieve good results with significantly higher levels of undersampling

Deep learning potentially provides faster imaging compared to current techniques by enabling higher acceleration factors (Fig. 1). Traditional methods show limited improvements in speed if compared to the latest DL techniques, as shown in the overview of image reconstruction methods. Such reconstruction methods achieve acceleration factors of 4 to 5 before image quality deteriorates too much [14]. Deep learning techniques demonstrate a significant improvement, allowing acceleration factors of up to 12 or more, depending on the intended use of the output, but their clinical efficacy has yet to be established [15].

**Fig. 2:**
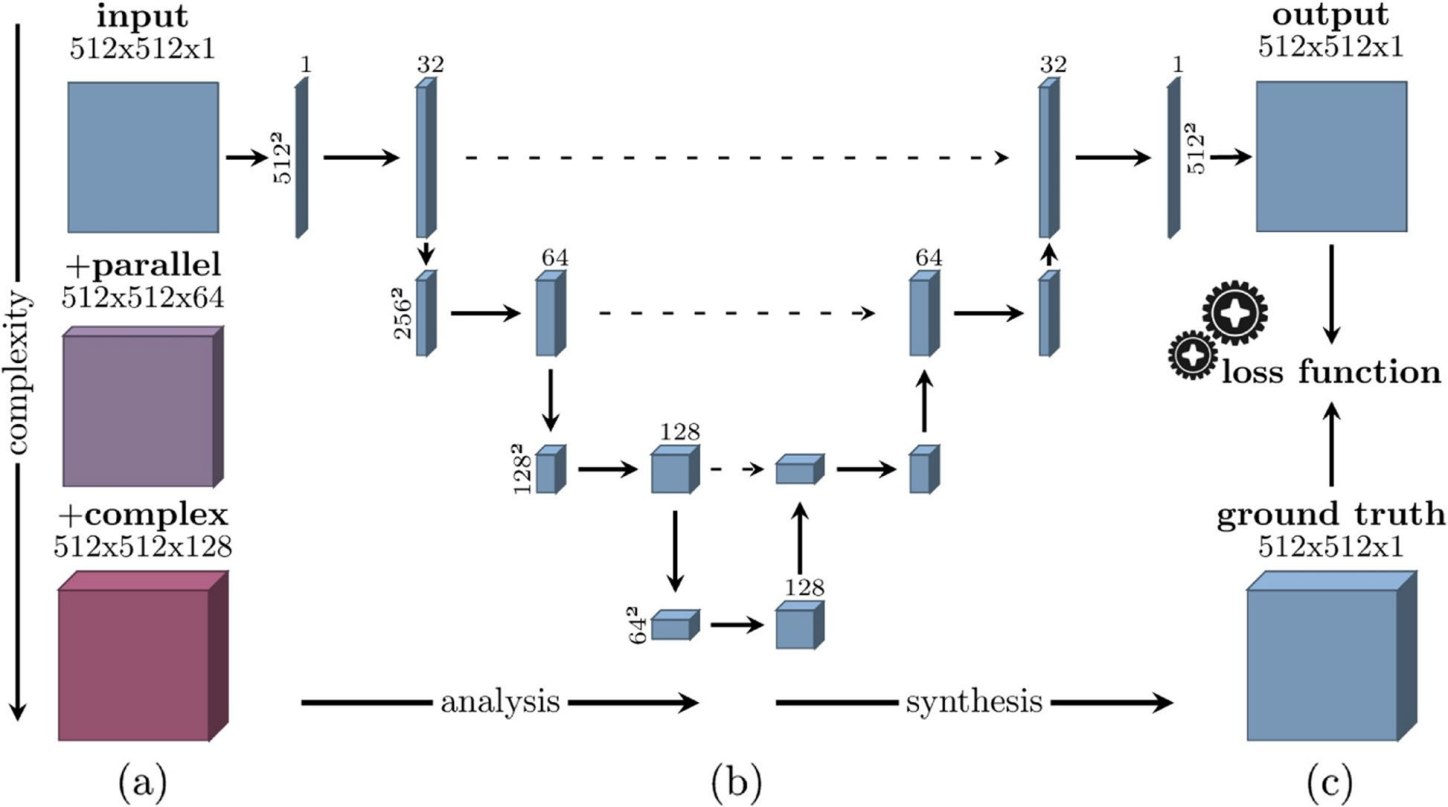
Diagram of a U-Net architecture. **a** The number of parameters in a network mainly depends on the input data. However, it can be expanded with parallel imaging and complex values. In this example, the input image size is 512 pixels square, which may be expanded with 64 sensitivity maps, or doubled to 128 when the complex data is separated into real and imaginary components. **b** Illustration of a U-Net architecture, where data is propagated through all paths in the network. The downsampling analyses the input, and the upsampling synthesizes an output. **c** The output is compared with the ground truth to calculate a score using a provided loss function

Reconstruction with DL involves transforming input data into an output image, which can be approached as an image-to-image task. Figure 2 illustrates a generic U-Net of a two-dimensional MR image reconstruction task, where a loss function is used to compare output to the ground truth. A U-Net in its basic form is limited to image-to-image reconstruction without any domain-specific knowledge, such as k-space, included. However, dual-domain networks allow both image and k-space to be utilized, and scan-specific methods can restore missing k-space [9, 16, 17].

MR image reconstruction today exists in a tension of three measures: image quality, robustness and the acceleration factor. Image quality can be improved by applying a cascade of networks, each of them evaluating the reconstruction independently [6]. However, these networks are shown to be sensitive to the sampling pattern, acceleration factor and noise level and deviations in the anatomy of unseen data [18, 19]. These factors may raise concerns about its robustness, and including controls for data consistency may reduce these sensitivities. Finally, various strategies exist for simulating the undersampling of fully sampled data, which may optimize the learning curve of a network in undersampled image reconstruction tasks. Many of these concepts have received considerable attention, and we will examine each of these in detail in the following subsections.

### Loss functions

The loss function is the function to be optimized during network training and is critical in any DL method. Commonly used loss functions are the mean-squared error (MSE) and structural similarity index metric (SSIM), which intend to maximize the similarity between a reconstructed image and its reference image [20]. Most DL methods are supervised: the loss function takes reference data (such as a reconstructed image from fully sampled k-space) as input to compute the loss. Yaman et al. [21] propose to take a fraction of their input k-space data as the input for their loss function as a form of self-supervision. They show that using sub-sampled k-space data as a reference does not significantly reduce the performance of the networks compared to using fully sampled images as a reference. This is useful when fully sampled reference data is unavailable and supervised methods cannot be trained. Zhou et al. [22] argue that fully sampled data is more expensive and more prone to motion and other accumulating errors. Instead, short undersampled acquisitions can be obtained in more ideal imaging conditions.

A loss function can also be a combination of loss functions. In addition to the MSE loss function, Yang et al. [23] also employ perceptual and adversarial loss functions. The perceptual loss is output by a pre-trained network that estimates human perceptual similarity (as opposed to a statistical similarity, such as SSIM). Adversarial loss is output by implementing a generative adversarial network (GAN), where two networks, a discriminator network and a generator network, contest each other: the discriminator aims to distinguish between an original reference image and a reconstruction output by the generator. Both networks optimize for their discriminative and generative ability, providing an adversarial loss.

### Undersampling strategies

An undersampled image reconstruction network can be trained by utilizing datasets with fully sampled images and partially eliminating k-space data to achieve an intended level of undersampling. The resulting output is then compared to the original data using a loss function with as goal of achieving similar quality. An ideal sampling strategy is one where the information of a ground truth image is best preserved given an amount of undersampling. Incoherence means information required for image reconstruction is distributed throughout k-space, and is a condition prescribed by compressed sensing. Deep learning networks are not well suited for aggregating spread-out information, and compressed sensing-based methods may not be preferable.

One common sampling strategy is variable density sampling. Similar to GRAPPA, the center of k-space is sampled more densely. This strategy is derived from compressed sensing, where better coherence is achieved using this method [3]. Defazio [24] proposes an equidistant sampling strategy, starting from the second k-line. This way, redundant sampling is avoided, while equidistant sampling allows for information to be localized within a small region, which is ideal for convolutional networks.

Adaptive sampling strategies and their underlying reconstruction algorithm are optimized in a data-driven manner. Bahadir et al. [25] propose a method that estimates a sampling density in k-space, describing which positions in k-space are most favorable given the underlying image reconstruction network. Aggarwal and Jacob [26] note that such an approach does not account for potential dependencies between sampling locations and, in their method, learns both the sampling pattern and the reconstruction problem jointly. Contrary to these arguments, Bakker et al. [27] show that their adaptive models learn to be explicitly nonadaptive. They hypothesize that adaptivity may compromise the model’s ability to capture relevant patterns in the data.

### Data consistency

Data consistency enforces that any reconstruction does not deviate from the underlying k-space data that was originally sampled. Schlemper et al. [6] introduce intermittently inserting data consistency layers in the architecture. This data consistency layer applies a penalty based on the difference between the sampled and output k-space value. This penalty is based on the assumption that the noise in k-space follows a normal distribution. In practice, k-space noise is more complex as noise accumulates from various sources around the scanner. Cheng et al. [28] propose to learn a more accurate representation of the noise distribution using their learned data consistency reconstruction method, giving more accurate penalties to data inconsistencies. In general, Hammernik et al. [29] show that networks with some form of data consistency substantially outperform networks without, implying that including DC is almost mandatory for MR image reconstruction. However, they note that the influence of data consistency wanes when acceleration factors are further increased, as there is less k-space to keep consistent.

### Parallel imaging

Parallel imaging gives additional information on the spatial location of the signal by using the sensitivity of each receive coil. The fastMRI public dataset included a challenge where participants submit their image reconstruction algorithms to compete in various competitions, and featured a competition where all coil information was combined into a single k-space volume [30]. In the following year, the next iteration of this challenge omitted this competition as the results were considered clinically irrelevant [31], suggesting that separate coil signal acquisitions cannot be combined without losing valuable information.

Deep learning-based methods that include multiple coil data as input are becoming more common. Sriram et al. [32] propose a method where the traditional parallel imaging technique GRAPPA is integral to their method. GRAPPA is a multi-coil parallel imaging technique where missing k-space lines due to undersampling are estimated for each coil using information obtained by densely sampling the center of k-space, known as calibration lines [1]. In their model, Sriram et al. use these calibration lines to estimate sensitivity maps, which are then applied to the multi-coil k-space input data. Wang et al. [33] perform parallel image reconstruction without integrating parallel imaging techniques but outperform these traditional methods using fewer calibration lines. Furthermore, they show relatively low sensitivity to the number of calibration lines. Leynes et al. [34] propose a method capable of calibrationless parallel image reconstruction by jointly solving the undersampled missing data of each coil and the reconstruction problem.

### Exploiting interslice correlations

Image reconstruction is most commonly performed on two-dimensional image slices, but consecutive slices in a multislice MR acquisition generally have strong interslice correlation. Various ways were suggested to leverage multislice correlations effectively. Pang and Zhang [35] propose a method which exploits this correlation using interpolated compressed sensing techniques [35]. Du et al. [36] take adjacent k-space slices as additional input and effectively interpolate this input to output the reconstruction of a single interpolated k-space slice. Xiao et al. [37] propose a method that uses deformable convolutions, jointly exploiting correlations among and within slices. They argue that deformable convolutions allow for efficient information extraction across neighboring slices, addressing complicated data redundancies more effectively than traditional fixed two-dimensional grids.

Interslice correlation is most effective using three-dimensional volumes; however, model training using such data size is very demanding in terms of model complexity and memory requirements. Du et al. [36] propose a model which claims to alleviate these concerns. It iterates across each dimension of a three-dimensional sample. Each iteration takes the information from the previous iteration as prior knowledge, thus minimizing the complexity of the model.

### Domains

Image space constrains the use of the previously discussed exploitable information, parallel imaging or interslice correlations, which may operate in k-space. Image reconstruction for undersampled acquisitions can be performed using image space to image space, k-space to k-space, or a hybrid k-space to image space, as their respective inputs and outputs. Most proposed methods expect image space as input, presumably as most available data is in image space, but this disregards the potential of utilizing the feature representations using different domains.

Under the premise of a “best of both worlds”, KIKI-net is a method operating on k-space first (K), image space second (I), and repeat (KI) in a hybrid fashion [16]. The first K-net is trained, and then the next I-net is trained using the output of the previous K-net. This process continues until the entire KIKI-net is fully trained. According to the authors, the I-nets were especially strong in restoring detailed structures but failed to remove aliasing artifacts. Instead, they further embellished the artifacts. Meanwhile, the K-nets removed artifacts successfully but had weaker structure restoring capabilities. Ran et al. [38] propose to process both image and k-space domains in parallel. They argue that processing sequentially, such as in KIKI and other variants, ignores possible interplay between domains.

Further motivated by the idea that employing more representations of the same data results in better performance, Wang et al. [39] propose IKWI-net, which includes the wavelet (W) transform domain. The authors argue that the wavelet domain is particularly effective in suppressing artifacts in smooth areas but may also embellish artifacts mistaken as real structures. Tong et al. [40] instead propose HIWDNet, which excludes the k-space domain, and uses entirely different network architectures for each domain.

Zhu et al. [8] propose a more genuine hybrid solution: automated transform by manifold approximation (AUTOMAP), which learns to reconstruct a spatial image directly from k-space. AUTOMAP performs a reconstruction without prior knowledge of mathematical transformations. In MR image reconstruction context, it implicitly learns a Fourier transform equivalent. AUTOMAP is criticized for its network complexity, and later variants reduce complexity by simply supplying the Fourier transform explicitly [41, 42].

### Complex numbers

Methods, such as AUTOMAP, use convolutional operations with only the magnitude of the originally complex-valued k-space data. The complex MR signal comprises a real and imaginary component. The imaginary component is phase-shifted by 90^◦^ and has independent and uncorrelated noise but is otherwise identical to the real component. The derived values magnitude and phase carry nonredundant information that underlines the relationship between the components. Most reconstruction methods that use k-space as input assume that the complex-valued signal components are independent, but this results in information loss due to their inherent interdependency [33]. This loss is made apparent by experiments performed by Cole et al. [43], which show two separate real- and imaginary-valued reconstructions as consistently superior to magnitude-based reconstructions.

Dedmari et al. [44] are the first to explore learning with complex-valued data using complex-valued convolutions. This was expanded by Wang et al. [33], who include parallel imaging. They claim their convolutional network using complex values achieves at least comparable performance with magnitude-valued convolutional networks while requiring half the network size. Feng et al. [45] propose dual-octave convolutions, which divide the real and imaginary components into low and high-frequency subcomponents. In k-space, low frequencies primarily contain image contrast information, and high frequencies hold the finer details. Processing these high and low frequencies separately is hypothesized to reduce spatial redundancy, making effective reconstructions easier. Finally, Terpstra et al. [46] identify that separating the loss function into two loss functions for magnitude and phase is noninferior in the absolute domain and superior in the complex domain.

## Deep learning MR image reconstruction training and evaluation

Understanding the previous topics may lead to making wise architectural choices, but researchers are restricted to using only the data they have. The output of DL learning methods is based on the data it was trained on, so dataset quality is crucial for their performance. Data is invariably scarce, and methods need to use available data efficiently. Dataset quality is determined by the number of images, the quality of the annotation of the target anatomy or pathology, standard of reference used, and other readily available metadata. Datasets are limited by time, technology, and regulations, while available compute and ingenuity primarily limit the methods that use them. These methods are typically modeled after blueprints of existing network architectures, heavily modified to make them suitable for MR image reconstruction. Evaluation of MR image reconstruction is nuanced, as reconstructions are expected to maintain general image quality and be robust in preserving any clinical pathologies. Results from the 2020 fastMRI challenge state that the top 3 methods, according to qualitative radiologist evaluation, still created hallucinatory features [31]. Meanwhile, statistical evaluation, such as the SSIM, shows results with up to 95% similarities to the ground truth. It is not implausible that relevant pathologies may still be hiding, or obscured by hallucinations, in the final dissimilar 5%. This implies a discordance between the image-derived statistical metrics for evaluating reconstructed images and the radiologist-defined diagnostic quality of an image. In other words, the diagnostic value of an image is unlikely defined by a single metric.

### Diagnostic quality

In the literature, it is common to demonstrate the capabilities of novel methods by comparing their output against established traditional and DL-based image reconstruction techniques. However, it is challenging to find metrics that directly correlate to the diagnostic quality of an image. Some image reconstruction methods position themselves as methods for artifact removal, implying that the undersampled acquisitions are images beset with artifacts [47]. Using such a definition, evaluation metrics such as MSE or SSIM cannot realistically quantify the quality of artifact removal. Blind evaluation performed by experienced radiologists is employed instead, where equalling or exceeding the diagnostic performance of radiologists while reducing acquisition time is the end goal [47].

Quantitative metrics may fail to reflect deficiencies in reconstructing fine details correctly. Zhao et al. [48] show insignificant differences between the SSIM values for images with and without lesions using various reconstruction methods, implying a weak correlation between SSIM and lesion detection capability. Mason et al. [49] compared a number of evaluation metrics and agreed with this observation, further noting similarly weak performance for root MSE. Instead, the less commonly used metrics, visual information fidelity [50], feature similarity index [51], and noise quality metric [52] demonstrate higher correlation with radiological assessment of image quality. However, these metrics have comparatively high computational costs.

These concerns of weak correlation do not align well with dataset projects such as the fastMRI project, which promotes statistical metrics for easier comparisons. Various editorials have created guidelines to improve the statistical reporting of studies that apply artificial intelligence to radiology [53–55]. Solutions which better quantify diagnostic image quality are critical for improving the feasibility of clinical integration of new methods.

### Robustness

Robustness is the ability of a method to not deteriorate in performance due to perturbations or other structural changes. For example, deviations between test and training data of brain MR images, which is likely given the high level of anatomy detail, will reduce network generalizability [19]. The datasets selected by new publications are hardly given motivation. Most datasets are collected from a single vendor. Methods developed using the fastMRI dataset, sourced from Siemens scanners, and applied to scans sourced from GE or Philips scanners show reduced performance [31]. This is highlighted by a study that applies stability tests on reconstruction methods to evaluate their performance after small data perturbations, such as simulating motion artifacts [56]. They show that a change of vendor should also be treated as a perturbation the learning algorithm is unprepared for. Knoll et al. [18] show that a mismatch in SNR has the most substantial influence on performance. Antun et al. [56] propose robustness tests with their instability tests. An example test is shifting the undersampling ratio slightly, which may lead to severe error during reconstruction. They conclude that a robust network would need to be retrained for different combinations of acquisition size, undersampling ratio, and other such parameters to decrease their sensitivity to various perturbations. Solutions to improve robustness with respect to specific artifacts are also proposed. Defazio et al. [57] introduce adversarial loss to combat banding artifacts, which are of particular note in low SNR regions. Their method is similar to a GAN, but the discriminator is focused on identifying banding, such that the generator is penalized for producing reconstructions with banding.

Due to the limited availability of raw k-space datasets, researchers often synthesize k-space data from image data using forward Fourier transforms. However, this approach has been labeled a “data crime”, a term coined by Shimron et al. [58]. They caution against using synthesized k-space data, which may lead to over-optimistic results. Deep learning networks benefit from data processed using vendor-specific hidden pipelines and will often produce optimistic results when compared to vendor-processed ground truth data. Undersampled image reconstruction networks are especially susceptible to these benefits, as the unprocessed undersampled acquisitions differ significantly from the processed and simulated k-space they are trained on.

### Data scarcity

Large datasets serve as the foundation for many DL models. Image reconstruction networks may benefit from more technically intricate datasets that offer complete k-space sampling. However, obtaining such datasets poses a formidable challenge. Public dataset releases are an important kickstart for new DL research. There are no public datasets including image-guided interventional MR data; unsurprisingly, we note a research gap in the application of undersampled image reconstruction networks developed for interventional radiology. Despite this, DL-based image reconstruction may be a more viable option for this field, as the enhanced reconstruction speeds can be more effectively leveraged, and the potential decline in image quality may be less detrimental.

Transfer learning provides a potential alleviation to the data scarcity problem. It is where a network trained on a task with large available datasets is transferred to another, usually with scarce datasets, by using network finetuning. Han et al. [47] demonstrate its potential by using networks pre-trained on radial computed tomography data for radial MR, then finetuning using radial MR data. Transfer learning has also shown promise by reconstructing images using a network initially trained to reconstruct large collections of natural images and brain MR images [59]. Huang et al. [19] observe that network finetuning could even be skipped if a network is pretrained using a large collection of generic MR images, rather than natural everyday images. If transfer learning proves effective in MR image reconstruction, even uncommon procedures which produce little data can be accelerated.

## Conclusions

Innovations in DL have given MR image reconstruction from undersampled acquisitions a boost. We explored the challenges posed by the complex nature of radiological input data and the importance of utilizing k-space information in undersampled image reconstruction. Key factors to consider in method design are those that leverage the potential of the available input data, such as inventive loss functions and versatile sampling strategies. Performance can be further enhanced if the data is enriched with additional information such as the original raw k-space data, coil sensitivity maps, and incorporating adjacent scans if from a multislice acquisition.

We discussed the difficulty of assessing the reconstructed image output for its diagnostic quality and robustness. We stress that while DL reconstruction output may provide high-quality images upon initial inspection, the reconstructions may also include hallucinations or omit small structural elements, limiting its diagnostic value. Moreover, it is important to ensure the robustness of MR image reconstruction models, given the sensitive nature of radiological data. One way to enhance robustness is by addressing the issue of data scarcity.

While notable steps have been taken in the right direction, it is crucial that new DL methods made for clinical use should be developed in collaboration with radiologists. These are to be supported with high-quality datasets, ideally open access and as multi-coil k-space data.

## Data Availability

Not applicable.

## Abbreviations

AUTOMAP: Automated transform by manifold approximation
DL: Deep learning
GAN: Generative adversarial network
GRAPPA: Generalized autocalibrating partial parallel acquisition
MR: Magnetic resonance
MSE: Mean squared error
SENSE: Sensitivity encoding
SNR: Signal-to-noise ratio
SSIM: Structural similarity index measure
